# Primary B cell lymphoma of the tongue base: a case report

**DOI:** 10.11604/pamj.2016.25.174.9746

**Published:** 2016-11-18

**Authors:** Achour Bechir, Achour Asma, Regaieg Haifa, Abdessayed Nesrine, Ben Youssef Yosra, Sriha Badreddine, Khelif Abderrahim

**Affiliations:** 1 Department of Hematology Farhat Hached Hospital, Sousse, Tunisia; 2 Department of Imaging Farhat Hached Hospital, Sousse, Tunisia; 3 Department of Pathology Farhat Hached Hospital, Sousse, Tunisia

**Keywords:** Non Hodgkin lymphoma, tongue, B-cell neoplasms

## Abstract

Primary non-Hodgkin’s lymphoma’s of the tongue is very rare and accounts for 1% of all malignant tumor of the oral cavity. Clinical features are non-specific ulcerative lesions that do not heal. In the literature, the majority of cases are diffuse large B cell type however, T cell phenotype also may occur. We describe a 77 years old man, who presented with an ulcerative mass in the left margin of the tongue the diagnosis diffuse large B cell lymphoma was confirmed. The patient is actually on treatment R-mini CEOP and has favorable evolution.

## Introduction

Non Hodgkin lymphoma represent the third most common group of malignant lesions of the oral cavity following squamous cell carcinomas and salivary gland neoplasms. Further only 1% of all lymphomas are primary oral cavity lymphoma. Non Hodgkin’s lymphoma’s of the tongue is extremely rare. It can arise from Waldeyer’s ring including the tonsils, nasopharyngeal lymphoid tissue, soft palate and base of tongue [1].

## Patient and observation

**Figure 1:**
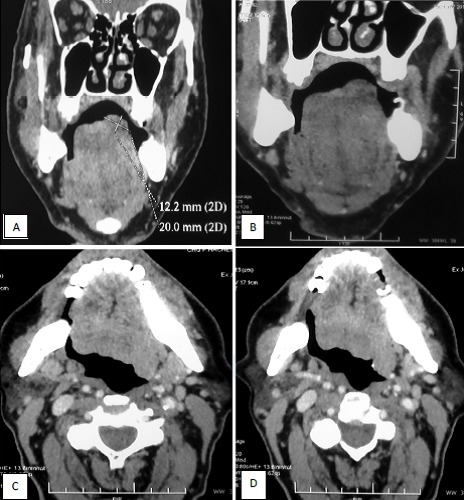
Tissue process of the root of the tongue: coronal (A) and axial (B); contrast-enhanced CT scan showing tissue process of the left margin of the tongue measuring 20x12 mm (C, D)

**Figure 2:**
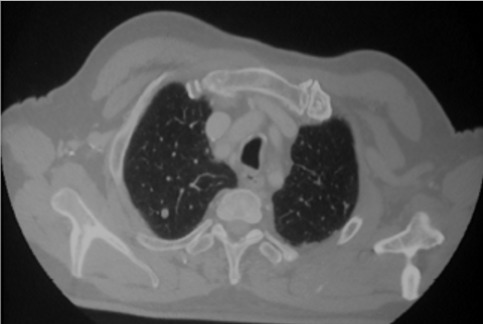
Axial CT scan in parenchymal window showing wright apicalpulmonary nodule of 6 mm

**Figure 3:**
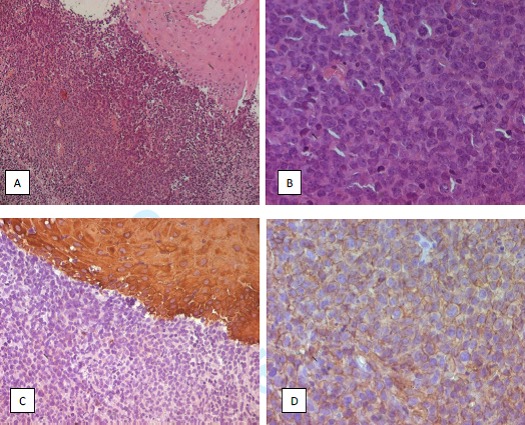
Histological examination of the tumour process: A) (HE: Gx100): lymphomatous diffuse involvement of the tongue; B) (HE: Gx400): lymphoid cells of large size with significant mitotic activity; C) (CK : Gx200): absence of expression of cytokeratin in tumour population; D) (CD20 : Gx400): diffuse and intense expression of CD20 in tumour cells

A 77 years old man presented with a history of odynophagia (dysphagia) and left sided pain of the tongue. Weight loss; night sweats and fever was reported. Local examination revealed an ulcerative lesion involving the left margin of the tongue and measured 2cm in diameter. The lesion was extensive to the right tonsil. Two cervical lymphadenopathy (jugular carotid) of 5 and 1 cm in diameter (Figure 1). Systematic examination including respiratory, cardiac, abdominal and central nervous system were normal except a hernia of the linea alba. The results of laboratory analyses obtained on admission were as follows: white cell count: 11700/ul; hemoglobin: 13.1 g/dl; platelet count: 223000/ul; lactate dehydrogenase: 213 u/l; calcium: 2.5 umol/l; blood urea nitrogen: 3.3 mg/l; creatinine: 46 umol/l; aspartate aminotransferase: 9 IU/l; alanine aminotrasferase: 8 IU/l. Computed tomography (CT) scan done after 2 cycles of chemotherapy (claustrophobic patient) revealed budding tissue process of the left margin of the tongue measuring 20*12 mm (picture 1), right apical pulmonar nodule of 6 mm. Calcified nodule of left apical pulmonar nodule of 5 mm. Small right laterotracheal lymphadenopathy of 8 mm of diameter (Figure 2). A biopsiy was performed. The histological examination showed a diffuse infiltration of great lymphoid cells with scarce cytoplasm, ovoid nucleus, sharp chromatin, great nucleus, numerous mitosis and apoptosis cells. Immunohistochemical evalution was positive for CD 20, CD 79a, CD30, CD15 and anti ALK suggestive of primary non-Hodgkin’s lymphoma large B cell type (Figure 3). He was staged as IVB. Bone marrow aspiration was normal. Chemotherapy consisted of the following regimen: Rituximab, Epirubicin, cyclophosphamide, vincristine and prednisone (R-miniCEOP), with favorable evolution and regression of the size of the lesion after 3 cycles of chemotherapy.

## Discussion

Primary malignant lymphoma of the tongue is very rare [1]. Elderly patients, especially those older than the sixth decade of life have been noted, and the male: female ratio is 5:3 [2]. In addition to the small number of cases reported in literature, few signs and symptoms [3] also characterize this disease, and the presenting symptoms were largely tongue masses [4] associated with progressive dyspnea and/or dysphagia [5] rather than constitutional symptoms such as weight loss, night sweats, and fever. Occasionally, the tumor may cause upper airway obstruction, which was the case of our patient. The tongue base itself appears to be an extremely unusual localization for isolated primary non-Hodgkin’s lymphoma [6]. After tissue biopsy, histopathological and immunohistochemical analyses is necessary to confirm a diagnosis of non-Hodgkin’s lymphoma, and imaging studies, either computed tomography (CT) or magnetic resonance imaging (MRI), to evaluate the laryngeal extension of the mass. Like lymphomas at other head and neck sites, non-Hodgkin’s lymphoma of the tongue also seem to be quite sensitive to both radiotherapy and chemotherapy [4, 6, 7]. The prognosis of lymphoma is related to the stage of tumor and the aggressiveness of the malignant cell type and the response to treatment [4, 7]. Our patient was treated with chemotherapy (type Rmini CEOP) and is programmed for radiotherapy and has favorable evolution.

## Conclusion

A localized non-Hodgkin’s lymphoma of the tongue base rarely occurs, and should be considered in the differential diagnosis of tongue base lesions. A proper clinical evaluation, histopathologic as well as immunohistochemical evaluation of biopsy specimen, are indeed essential to improve the management and the prognosis of this disease.
